# A novel approach for breast cancer treatment: the multifaceted antitumor effects of rMeV-Hu191

**DOI:** 10.1186/s41065-024-00337-9

**Published:** 2024-09-28

**Authors:** Xiao-Yu Zheng, Yao Lv, Ling-Yan Xu, Dong-Ming Zhou, Lan Yu, Zheng-Yan Zhao

**Affiliations:** 1 Department of Ophthalmology, Children’s Hospital, Zhejiang University School of Medicine, National Clinical Research Center for Child Health, Hangzhou, China; 2 Department of Gastroenterology, Children’s Hospital, Zhejiang University School of Medicine, National Clinical Research Center for Child Health, Hangzhou, China; 3 The Second Affiliated Hospital of Jiaxing University, Jiaxing, China; 4 Children’s Hospital, Zhejiang University School of Medicine, National Clinical Research Center for Child Health, Hangzhou, China; 5 Department of Child Health Care, Children’s Hospital, Zhejiang University School of Medicine, National Clinical Research Center for Child Health, Hangzhou, China; 6 No. 3333 Binsheng Road, Hangzhou, Zhejiang Province 310052 China

**Keywords:** Breast cancer, Recombinant measles Hu191 vaccine strain, Oncolytic virus, Apoptosis, Senescence, Proliferation inhibition, Oxidative stress, Lipid homeostasis

## Abstract

**Background:**

The therapeutic potential of oncolytic measles virotherapy has been demonstrated across various malignancies. However, the effectiveness against human breast cancer (BC) and the underlying mechanisms of the recombinant measles virus vaccine strain Hu191 (rMeV-Hu191) remain unclear.

**Methods:**

We utilized a range of methods, including cell viability assay, Western blot, flow cytometry, immunofluorescence, SA-β-gal staining, reverse transcription quantitative real-time PCR, transcriptome sequencing, BC xenograft mouse models, and immunohistochemistry to evaluate the antitumor efficacy of rMeV-Hu191 against BC and elucidate the underlying mechanism. Additionally, we employed transcriptomics and gene set enrichment analysis to analyze the lipid metabolism status of BC cells following rMeV-Hu191 infection.

**Results:**

Our study revealed the multifaceted antitumor effects of rMeV-Hu191 against BC. rMeV-Hu191 induced apoptosis, inhibited proliferation, and promoted senescence in BC cells. Furthermore, rMeV-Hu191 was associated with changes in oxidative stress and lipid homeostasis in infected BC cells. In vivo, studies using a BC xenograft mouse model confirmed a significant reduction in tumor growth following local injection of rMeV-Hu191.

**Conclusions:**

The findings highlight the potential of rMeV-Hu191 as a promising treatment for BC and provide valuable insights into the mechanisms underlying its oncolytic effect.

**Supplementary Information:**

The online version contains supplementary material available at 10.1186/s41065-024-00337-9.

## Introduction

Breast cancer (BC), the most common malignant tumor in women worldwide, has a high mortality rate. According to the World Health Organization (WHO), there will be 19.3 million diagnosed cases of BC by 2025, and an estimated 627,000 women died from BC in 2018 [1]. In China, the incidence of BC has continued to rise over the past 30 years, with more than 410,000 new cases expected in 2020 [2]. BC remains a major public health concern.

BC is a heterogeneous disease that requires a multidisciplinary approach to treatment. Over the past century, treatment approaches have evolved significantly [3]. These approaches include local interventions such as surgery and radiotherapy, as well as systemic therapies like endocrine therapy, chemotherapy, anti-HER2 therapy, bone stabilizers, immunotherapy, etc [4, 5]. Despite these advancements, approximately 20–30% of patients eventually develop advanced BC with distant metastases [6]. The limited efficacy of current treatments often results in patients dying from complications, highlighting the urgent need for novel, effective, and safe therapeutic techniques, particularly for tumors resistant to conventional treatments [7, 8].

Oncolytic virus (OV) therapy offers a promising new therapeutic option for BC patients. OVs selectively infect and kill tumor cells, inducing systemic antitumor immunity with minimal impact on healthy tissue. The oncolytic effect of viruses was first observed in the early 19th century when some patients with hematological malignancies experienced spontaneous regression of cancer following viral infection [9]. OVs can be naturally tumor-targeted or engineered to enhance tumor specificity. Various OVs have been researched for BC treatment, including measles virus, adenovirus, echovirus, herpes simplex virus, Newcastle disease virus, cowpox virus, etc [10–12].

Among these, measles virus vaccine strains are reasonable candidates for oncolytic therapy, as wild-type strains are too dangerous to use in cancer treatment. The Hu191 vaccine strain has been used extensively in China for over 60 years, confirming its safety and reliability. Our team has successfully engineered the recombinant measles Hu191 vaccine strain (rMeV-Hu191) using a reverse genetics system [13]. This strain can be serially passaged and replicated to high titers. Previous studies have shown promising oncolytic effects of rMeV-Hu191 in various cancers, including gastric, colorectal, pancreatic, esophageal carcinoma, and nephroblastoma [14–17]. We aim to explore the oncolytic activity of rMeV-Hu191 against BC to develop novel therapeutic approaches for this malignancy.

Investigations into the mechanisms of OVs have shown significant differences between various viruses and tumors. These mechanisms include cell cycle arrest, apoptosis, autophagy, and immunogenic death, etc [18–22]. However, the oncolytic mechanism of rMeV-Hu191 in BC remains to be examined.

The mammary gland is rich in lipids, and the development and progression of BC are closely linked to lipid metabolism disorders, including abnormal lipid accumulation and changes in cellular lipid profiles [23, 24]. Clinical studies have demonstrated that blood lipid abnormalities worsen after chemotherapy in BC patients and that lipid levels can predict BC prognosis [23]. Modulating lipid metabolism can affect the growth of BC cells and their response to drugs [25, 26]. Whether rMeV-Hu191 affects lipid homeostasis in BC has not been studied.

Herein, we elucidated the antitumor effect of rMeV-Hu191 on BC cells in vitro and in vivo. We investigated the roles of apoptosis, senescence and cell proliferation in virotherapy, and revealed the impact of the measles virus on oxidative stress and lipid homeostasis in BC cells. These findings lay a scientific foundation for the further development of oncolytic therapies for BC patients.

## Materials and methods

### Cell lines and cultures

Vero (African green monkey kidney) was purchased from the American Type Culture Collection (ATCC). Human BC cell lines including MDA-MB-231, MDA-MB-468 and BT549 were obtained from the Cell Bank of Type Culture Collection Chinese Academy of Sciences. The cells were cultured in cell culture flasks (Corning) in Dulbecco’s modified Eagle’s medium (DMEM) (Gibco) or RPMI 1640 medium (BT549). These media were supplemented with 10% inactivated fetal bovine serum (FBS) (Gibco), 1% L-glutamine, and 1% penicillin/streptomycin (Gibco). The cultures were incubated in a humidified atmosphere with 5% CO_2_ at 37 °C.

### Amplification of measles virus vaccine strain

The virus stock was generated by infecting VERO cells cultured in Opti-MEM (Gibco) at a multiplicity of infection (MOI) of 0.1. About 24–36 h later, after the cells had fused into syncytia, the freeze-thaw procedure was repeated three times on dry ice. The samples were centrifuged (4 °C, 4500 g, 10 min), and the supernatants were collected. The virus stocks were quantified using a plaque-forming unit (PFU) assay on VERO cells.

### Detection of CD46 and Nectin-4 expression levels

A total of 1 × 10^6^ cells were analyzed by flow cytometry (BD Biosciences) following the protocols of the antibody identification kit. Briefly, after culturing for 48 h, the BC cells were digested with 0.25% trypsin without EDTA (Gibco, 15090-046; 1mL/T25) for 5 min. The cells were centrifuged at 800 g for 3 min at 25 °C, then resuspended and washed twice with sterile PBS. The BC cells were blocked with 2% BSA (in 1× PBS) on ice for 40 min, then 10 µl/sample of PE-Mouse anti-human IgG (BD 555787) was added, with PE mouse anti-human CD46 antibody (BD 564252) or PE mouse anti-human Nectin-4 antibody (BD 564252). The samples were gently mixed with a pipette and incubated on ice in the dark for 40 min. Then the cells were centrifuged again under the same conditions, washed twice with PBS, and resuspended in 600 µl PBS. Flow cytometry analysis was performed using the PE channel.

### Cell viability assays

5 × 10^3^ cells /well of BC cells seeded in 96-well plates were infected with multiple MOIs (0, 0.1, 0.5, 1, 5, 10) of rMeV-Hu191, and cell viability was quantified every 24 h with a Cell Counting Kit-8 (CCK8, TargetMol). For assessment, cells were cultured with 100 µl working medium consisting of 90 µl DMEM and 10 µl reagent, for 30 min at 37 °C, and the absorbance at 450 nm was measured using a multifunctional microporous plate detector (TECAN-SPARK).

### Cytotoxicity of rMeV-Hu191 in vitro

The BC cells (2 × 10^5^ cells/well) seeded in 6-well plates were infected with rMeV-Hu191 at multiple MOIs (0, 0,1, 0.5, 1, 5, and 10). After 96 h post-infection, the medium was removed and the cells were fixed with 4% paraformaldehyde at room temperature for 2 h. Paraformaldehyde was discarded, and the remaining cells were stained with 0.1% crystal violet for 7 min, rinsed with water, and photographed under a scanner (Canon).

### Cell death analysis by flow cytometry

The BC cells (2 × 10^5^ cells/well) seeded in 6-well plates were exposed to rMeV-Hu191 at an MOI of 0.1, with or without 50 µM Z-VAD-FMK (A1902, ApexBio), and then harvested for 48 and 72 h. To assess apoptosis, flow cytometry was conducted using an annexin V-fluorescein isothiocyanate (annexin V-FITC) apoptosis detection kit from BD Bioscience following the manufacturer’s protocol. Briefly, following rinsing with PBS, 1 × 10^5^ cells were resuspended in 100 µl of binding buffer, and then treated with 5 µl each of propidium iodide (PI) and annexin V-FITC for 15 min in the dark at room temperature. Analysis was performed with a flow cytometer (BD).

### Western blot

Proteins were extracted using RIPA lysis buffer (Beyotime Biotechnology) and quantified using a BCA detection kit (Beyotime Biotechnology) according to the manufacturer’s instructions. Equal amounts of protein were loaded onto SDS-PAGE gels and electrophoretically transferred onto PVDF membranes (Bio-Rad). The membranes were then blocked with 5% nonfat milk in Tris-buffered saline for 1 h at room temperature. The membranes were then incubated with specific primary antibodies overnight at 4 °C, followed by incubation with appropriate horseradish peroxidase-conjugated secondary antibodies for 1 h at room temperature. Protein signals were visualized using an enhanced chemiluminescence reagent (Biological Industries) and captured using a chemiluminescent detection system (Gene X). Reagents for Western blot analysis: anti-caspase-3 (CST, 9665 S, 1:1000), anti-caspase-7 (CST, 9492, 1:1000), anti-PARP (CST, 9542 S, 1:1000), anti-β-actin (CST, 4970 S, 1:5000), anti-CDK4 (Abcam, ab108357, 1:2000), anti-cyclin D1 (Abcam, ab134175, 1:2000), anti-β-tubulin (CST, 2146 S, 1:10000), anti-measles nucleoprotein (anti-MV-N, Abcam, ab106292, 1:5000), anti-GAPDH (CST, 5174 S, 1:5000).

### 5-Ethynyl-2′-deoxyuridine (EdU) cell proliferation assays

2 × 10^5^ cells/well of BC cells seeded in 6-well plates were infected with rMeV-Hu191. Cell proliferation was measured using a BeyoClick™ EdU-594 Cell Proliferation Assay Kit (Beyotime Biotechnology, #C0078S). 20 µM pre-warmed 2X EdU working solution was added, and cells were incubated for 4 h. Cells were then fixed with 1 mL of 4% paraformaldehyde for 15 min, washed three times, and permeabilized with 0.3% Triton X-100 in PBS for 10 min. After two washes, the Click reaction solution was added (0.5 mL per well) and incubated in the dark for 30 min. Nuclei were stained with 1X Hoechst33342 solution for 10 min, followed by three washes. Fluorescence detection was performed using a Zeiss Observer Z1 fluorescence microscope, using the DAPI channel for Hoechst 33,342 and the RFP channel for EdU-labeled cells.

### Immunofluorescence

The cells were planted in glass coverslip-bottomed chambers (Corning), and treated with vehicle or oncolytic measles at an MOI of 0.5 for 36 h. Following two rinses with PBS, the cells were fixed in 4% paraformaldehyde at 25 °C for 20 min and subsequently permeabilized with 1% Triton X-100 in PBS at 25 °C for 10 min. The primary antibodies used were anti-MV-N (Abcam, ab106292, 1:500) and anti-Ki67 (Abcam, ab15580, 1:500). The cells were washed with PBS three times and incubated with a fluorescent secondary antibody at room temperature for 1 h. After washed with PBS, the cells were stained with DAPI (Beyotime Biotechnology, C1002) for 10 min. Then, the cells were observed under Zeiss Observer Z1 fluorescence microscopy.

### Senescence-associated β-galactosidase (SA-β-gal) staining

The BC cells (2 × 10^5^ cells/well) seeded in 6-well plates, were placed with glass crawlers in advance, and then were infected with rMeV-Hu191 at an MOI of 0.1. At 24–36 h post-treatment, the cells were fixed with 4% paraformaldehyde at room temperature for 20 min, washed with PBS twice, and incubated at 37 °C with fresh SA-β-gal stain solution (Beyotime Biotechnology) for 12 h. After staining, the cells were washed in PBS and then compressed between glass slides for observation under a light microscope (Zeiss Observer Z1).

### Total intracellular ROS detected by fluorescence microscopy

Intracellular ROS signaling was tested by an ROS assay kit (Beyotime Biotechnology, #S0033). After treated with rMeV-Hu91 at an MOI of 0.5 for 36 h, the cells were incubated with 10 µM 2’,7’-dichlorodihydrofluorescein diacetate (DCFH‐DA) for 20 min at 37 °C and then were washed with DMEM three times. Then, the fluorescence distribution was detected by a Zeiss Observer Z1 fluorescence microscope.

### Flow cytometry for intracellular ROS measurement

The treatment of rMeV-Hu191 was described as above. After being infected with rMeV-Hu191 for 24–36 h, the BC cells were dispersed into single cells by 0.25% Trypsin-EDTA (Gibco) and incubated with 10 µM DCFH-DA for 20 min at 37 °C. Then, the fluorescence intensity and proportion of positive cells were measured in the FITC channel by a flow cytometer (Navios, Beckman Coulter, USA), and the results were analyzed using FlowJo 10.0 software (Tree Star Inc, USA).

### Detection of mitochondrial membrane potential (MMP)

MMP was detected with a JC-1 Kit (Beyotime Biotechnology, #C2006). In brief, after treatment with or without rMeV-Hu191, the BC cells were harvested with trypsin and suspended in PBS. JC-1 staining solution was obtained by diluting 50 µL JC-1 (200X) with 8 mL ultrapure water, and then 2 mL JC-1 staining buffer (5X) was added. The BC cells were stained with 1 mL JC-1 staining working solution and incubated at 37 °C for 20 min. JC-1 staining buffer (5X) was diluted with distilled water (1:4) to prepare a 1X buffer. After incubation, the supernatant was removed, and cells were washed twice with 1X JC-1 staining buffer. Then 2 mL cell culture medium was added. Finally, each group was analyzed with a Zeiss Observer Z1 fluorescence microscope in the GFP and cyc3 channels. The images were analyzed using ImageJ software (Version 2.0.0).

### RNA isolation and reverse transcription quantitative real-time PCR (RT-qPCR)

BC cells (2 × 10⁵ per well) were seeded in 6-well plates and infected with rMeV-Hu191 for 48 h. Total RNA was extracted using TRIzol (Thermo Fisher). Cells were lysed with 1 mL TRIzol per 10 cm², incubated for 5 min, then mixed with 500 µL chloroform per 1 mL TRIzol and centrifuged at 12,000 g for 15 min at 4 °C. The aqueous phase was mixed with 500 µL isopropanol per 1 mL TRIzol and centrifuged at 12,000 g for 10 min at 4 °C. The RNA pellet was washed with 70% ethanol, air-dried, and dissolved in double-distilled water. Then the RNA was reverse-transcribed using Prime ScriptTM RT Master Mix (Takara). Quantitative real-time PCR was performed using TB Green^®^ Premix Ex Taq™ (Takara) on a real-time PCR system (ABI-7500 Step-One Plus). The relative expression was normalized to that of GAPDH by the 2-ΔΔCt method. The primer sequences are listed in Table S1.

### Transcriptome sequencing

RNA samples (triplicate) from control or rMev-Hu191–treated MDA-MB-231 (MOI = 0.1 each for 48 h) were isolated using TRIzol. For RNA library preparation, PolyA-selection was used with a target fragment size of 300–500 bp. PCR products were cleaned with AMPure XP beads by mixing 65 µl of beads with 100 µl of PCR product, incubating, and separating on a magnetic rack. Beads were washed with ethanol, air-dried, and RNA was eluted with NF-H2O. Libraries were pooled and sequenced using the Illumina NovaSeq 6000 platform. Data quality was evaluated, and libraries were rebuilt if needed. Data processing included RNA-seq analysis, differential expression analysis with DESeq2 (v1.4.5), and functional enrichment analysis using the BGI Dr.Tom system.

### Immunohistochemistry

The tumor tissues were fixed in 4% formalin overnight and embedded in paraffin. Slides were treated with xylene I and II for 15 min each, then rehydrated with ethanol gradients (100%, 85%, 75%) and distilled water. Antigen retrieval was performed by heating slides with citrate buffer (95–99 °C, 10 min), EDTA (95–99 °C, 15 min), or TE buffer (95–99 °C, 18 min), followed by cooling. Pepsin digestion was conducted at 37 °C for 10 min. The tumor slices were stained with the primary anti-Ki67 (Abcam, ab15580, 1:500) at 4 °C overnight. After washed with PBS three times, all slices were incubated with the appropriate concentrations of biotinylated secondary antibodies and imaged using DAB solution. Finally, the slices were observed under a microscope (Zeiss Observer Z1).

### Statistical analysis

Each experiment was conducted in triplicate, and three independent experiments were performed. Data analyses were conducted using one-way ANOVA followed by the Bonferroni post hoc test. Pairwise comparisons were performed using a two-tailed unpaired t-test. Survival curves were plotted using Kaplan-Meier analysis with the log-rank Mantel-Cox test. All statistical analyses were performed using GraphPad Prism 9.4. Quantification data were represented as mean ± standard error, with p-value thresholds set at *, 0.05; **, 0.01; ***, 0.001; and ****, 0.0001.

## Results

### BC cells express receptors for measles virus infection

The expression levels of CD46 and Nectin-4, which are the main receptors for measles virus, were assessed in the BC cell lines MDA-MB-231, MDA-MB-468, and normal human umbilical vein endothelial cells (HUVEC) using flow cytometry. CD46 was highly expressed in 100% of MDA-MB-231 cells, 99.7% of MDA-MB-468 cells, and 99.4% of HUVEC cells. Nectin-4 was barely detected in MDA-MB-231 and HUVEC cells, but abundantly expressed in 97.9% of MDA-MB-468 cells (Fig. S1). These findings indicate that measles virus can inherently infect the BC cell lines MDA-MB-231 and MDA-MB-468.

### rMeV-Hu191 virus reduces the viability of BC cells

**Fig. 1:**
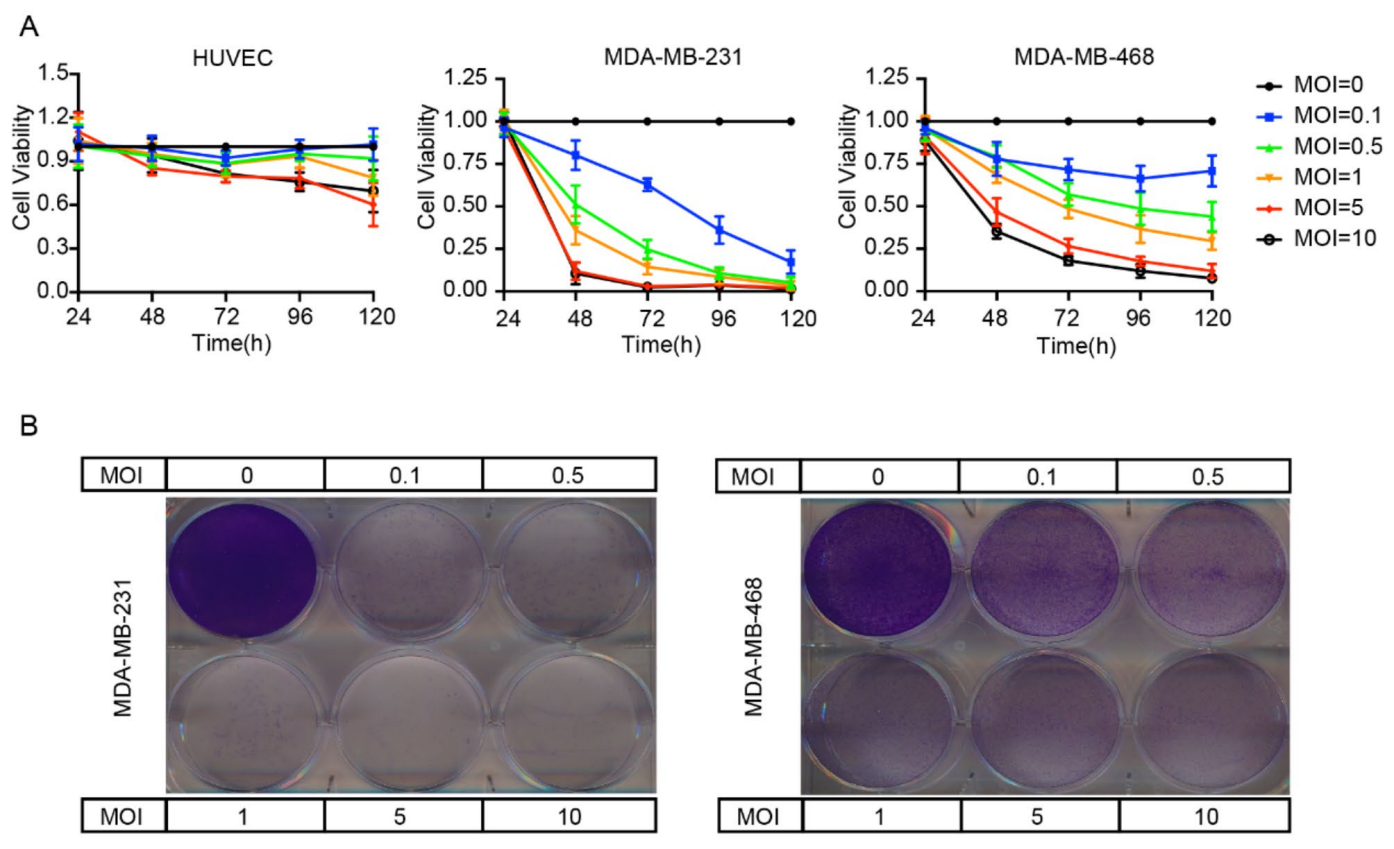
Infection and cytopathic effects of rMeV-Hu191 virus on BC cell lines. (A) Assessment of cell viability in BC cells infected with rMeV-Hu191 at different multiplicity of infections (MOIs) over time using the CCK8 assay. Data are expressed as mean ± standard error of three replicate experiments. (B) Evaluation of the cytotoxicity in BC cells after rMeV-Hu191 infection through crystal violet staining

The CCK8 assay demonstrated that rMeV-Hu191 significantly decreased the viability of MDA-MB-231 and MDA-MB-468 BC cells compared to the control. The antitumor effects against BC cells were both MOI- and time-dependent (Fig. 1A). Additionally, crystalline violet staining revealed a significant reduction in the number of viable BC cells at 96 h post-infection, along with the formation of distinct virus plaques, also showing MOI dependency. Notably, at equivalent MOIs, rMeV-Hu191 exhibited a more pronounced cytopathic effect on MDA-MB-231 cells than on MDA-MB-468 cells, as shown in Fig. 1B.

### rMeV-Hu191 stimulates oxidative damage and caspase-dependent apoptosis in BC cells

**Fig. 2:**
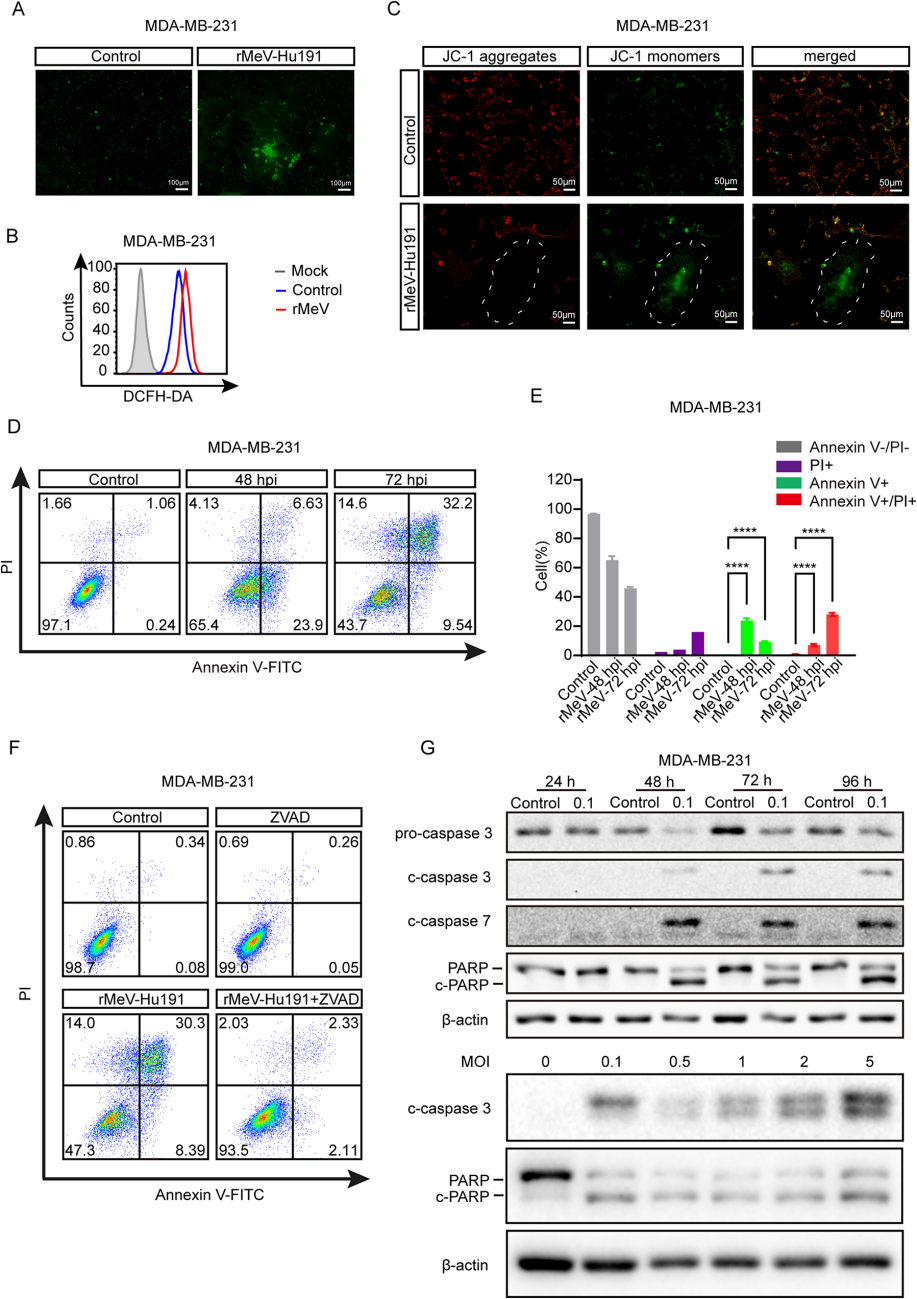
Oxidative stress damage and apoptosis induced by rMeV-Hu191 in BC cells. (**A**) Measurement of ROS production in BC cells using 2’,7’-dichlorodihydrofluorescein diacetate (DCFH‐DA) staining. (**B**) Determination of cellular ROS levels by flow cytometry with DCFH-DA staining. (**C**) Detection of mitochondrial membrane potential (MMP) changes in rMeV-Hu191-infected BC cells by JC-1 staining. (**D**) Flow cytometry analysis of apoptotic cells in MDA-MB-231 cells following rMeV-Hu191 infection, as assessed by annexin V-FITC/PI staining. (**E**) Statistical analysis of apoptotic cell proportions in MDA-MB-231 cells post rMeV-Hu191 infection, with data represented as mean ± standard error from three independent experiments. (**F**) Reversal of rMeV-Hu191-induced apoptosis in MDA-MB-231 cells by Z-VAD-FMK treatment. (**G**) Western blot analysis of cleaved caspase 3 (c-caspase 3), cleaved caspase 7 (c-caspase 7), and cleaved PARP (c-PARP) expressions induced by rMeV-Hu191 virus at different time points and various MOIs. hpi: hours post-infection

After treatment with rMeV-Hu191 at an MOI of 0.5 for 36 h, DCFH-DA staining revealed stable green fluorescence localized within the syncytial regions of both MDA-MB-231 and BT-549 cells, indicative of intracellular ROS production (Fig. 2A and Fig. S2A). Flow cytometry analysis showed a rightward shift of ROS generation in the rMeV-Hu191-infected groups compared to the control group (Fig. 2B and Fig. S2B). In addition, JC-1 staining demonstrated rMeV-Hu191-induced downregulation of MMP, indicated by a decreased red/green fluorescence ratio in the infected group, suggesting mitochondrial damage and oxidative stress (Fig. 2C and Fig. S2C).

Infected with rMeV-Hu191 at an MOI of 0.1 resulted in apoptosis rates of 30.5% at 48 h and 41.7% at 72 h in MDA-MB-231 cells, while MDA-MB-468 cells showed apoptosis rates of 14.3% at 48 h and 39.7% at 72 h (Fig. 2D and Fig. S2D). Repeated three times using MDA-MB-231 cells, the experiments consistently demonstrated a significant increase in apoptosis rates with rMeV-Hu191 infection (*p* < 0.01), with early apoptosis predominantly observed at 48 h and late apoptosis at 72 h (Fig. 2E). Furthermore, the apoptosis inhibitor Z-VAD-FMK significantly reversed the rMeV-Hu191-induced apoptosis in MDA-MB-231 cells (Fig. 2F). The expression levels of cleaved caspase 3, cleaved caspase 7 and cleaved PARP increased over time and with higher MOI (Fig. 2G).

### rMeV-Hu191 inhibits BC cell proliferation and induces senescence

**Fig. 3:**
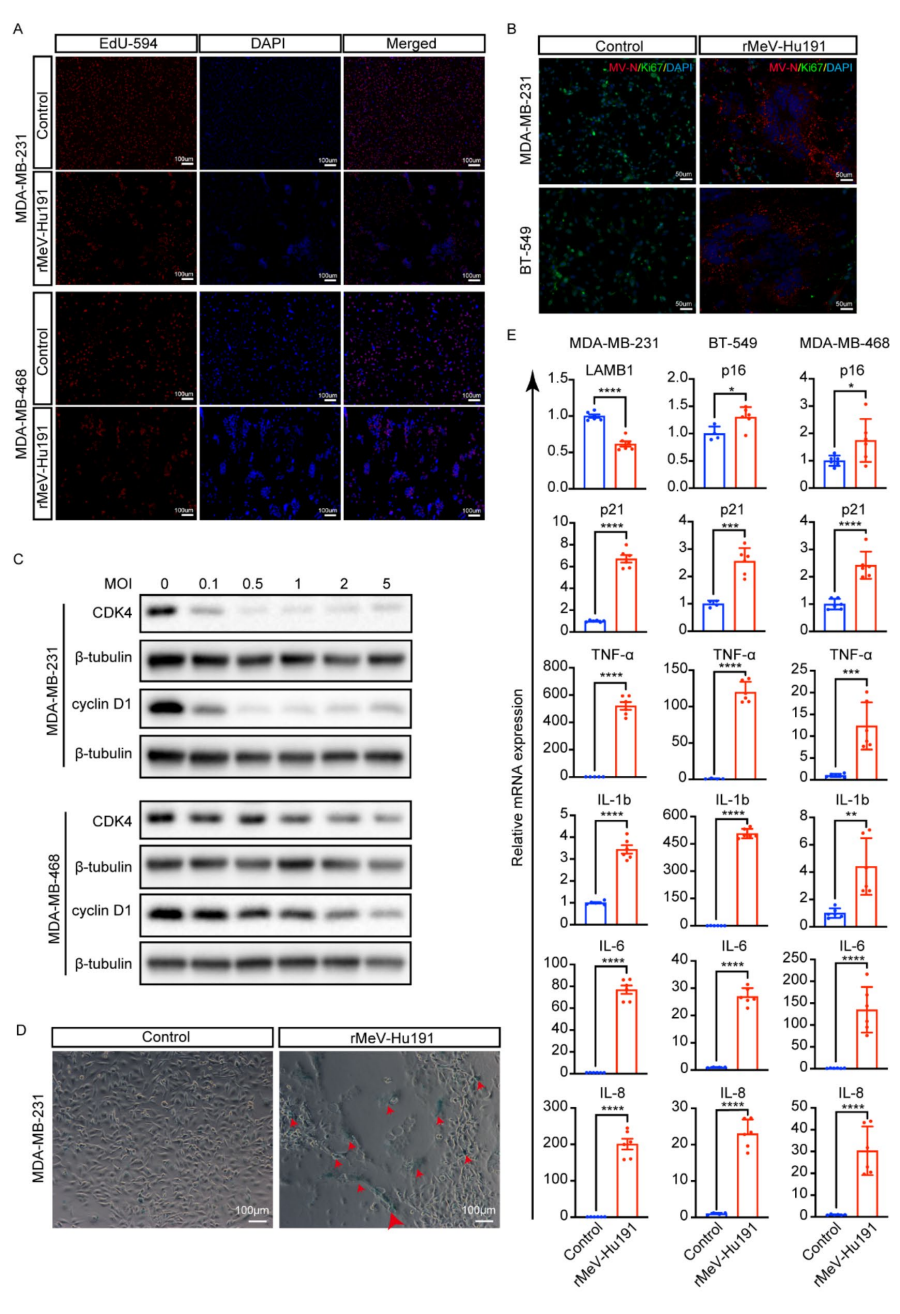
Proliferation inhibition and senescence of BC cells infected with rMeV-Hu191. (**A**) Evaluation of cell proliferation in MDA-MB-231 and MDA-MB-468 cells post rMeV-Hu191 infection using an EdU staining assay, with DAPI for nuclear labeling. (**B**) Assessment of proliferative activity in cells following rMeV-Hu191 infection using Ki67 immunofluorescence staining assay in MDA-MB-231 and BT-549 cells. (**C**) Detection of CDK4 and cyclin D1 by Western blot analysis after rMeV-Hu191 infection. (**D**) Evaluation of the senescence status of BC cells post rMeV-Hu191 infection using senescence-associated β-galactosidase (SA-β-gal) staining assay. The red arrowhead indicates SA-β-gal staining-positive senescent cells. (**E**) Detection of senescence markers and senescence-associated secretory phenotype (SASP) factors using reverse transcription quantitative real-time PCR (RT-qPCR) method after rMeV-Hu191 infection in BC cell lines. *p < 0.05, **p < 0.01, ***p < 0.001, ****p < 0.0001

EdU, a thymine nucleoside analog, is incorporated into the nucleus during cell proliferation and appears red upon EdU staining. Following infection with rMeV-Hu191 at an MOI of 2 for 24 h, a decrease in the number of proliferating cells exhibiting red fluorescence was observed in MDA-MB-231 and MDA-MB-468 BC cells (Fig. 3A). Ki-67, a nuclear protein expressed during all active cell cycle phases, showed significantly reduced expression in the syncytial regions of MDA-MB-231 and BT-549 BC cells after infection with rMeV-Hu191 at an MOI of 2 for 24 h, indicating inhibited BC cell proliferation (Fig. 3B). Additionally, when MDA-MB-231 and MDA-MB-468 BC cells were infected with rMeV-Hu191 at MOIs of 0.1, 0.5, 1, 2, and 5 for 24 h, the expression levels of CDK4 and cyclin D1 decreased in an MOI-dependent manner (Fig. 3C).

The SA-β-gal staining assay revealed increased SA-β-gal activity in the syncytial regions of senescent BC cells 48 h post-infection with rMeV-Hu191 at an MOI of 0.1 (Fig. 3D). The absence of the nuclear marker lamin B1 (LAMB1) and the aggregation of the cell cycle proteins p16 and p21 are important typical senescence markers. Additionally, most senescent cells exhibit a senescence-associated secretory phenotype (SASP), which includes interleukins (such as IL-1b, IL-6, and IL-8) and tumor necrosis factor (TNFα). The relative mRNA levels of p16, p21, LAMB1 (MDA-MB-231 cells do not express p16 [27]), and SASP components were significantly altered post-infection (Fig. 3E).

### rMeV-Hu191 disrupts lipid homeostasis in BC cells

**Fig. 4:**
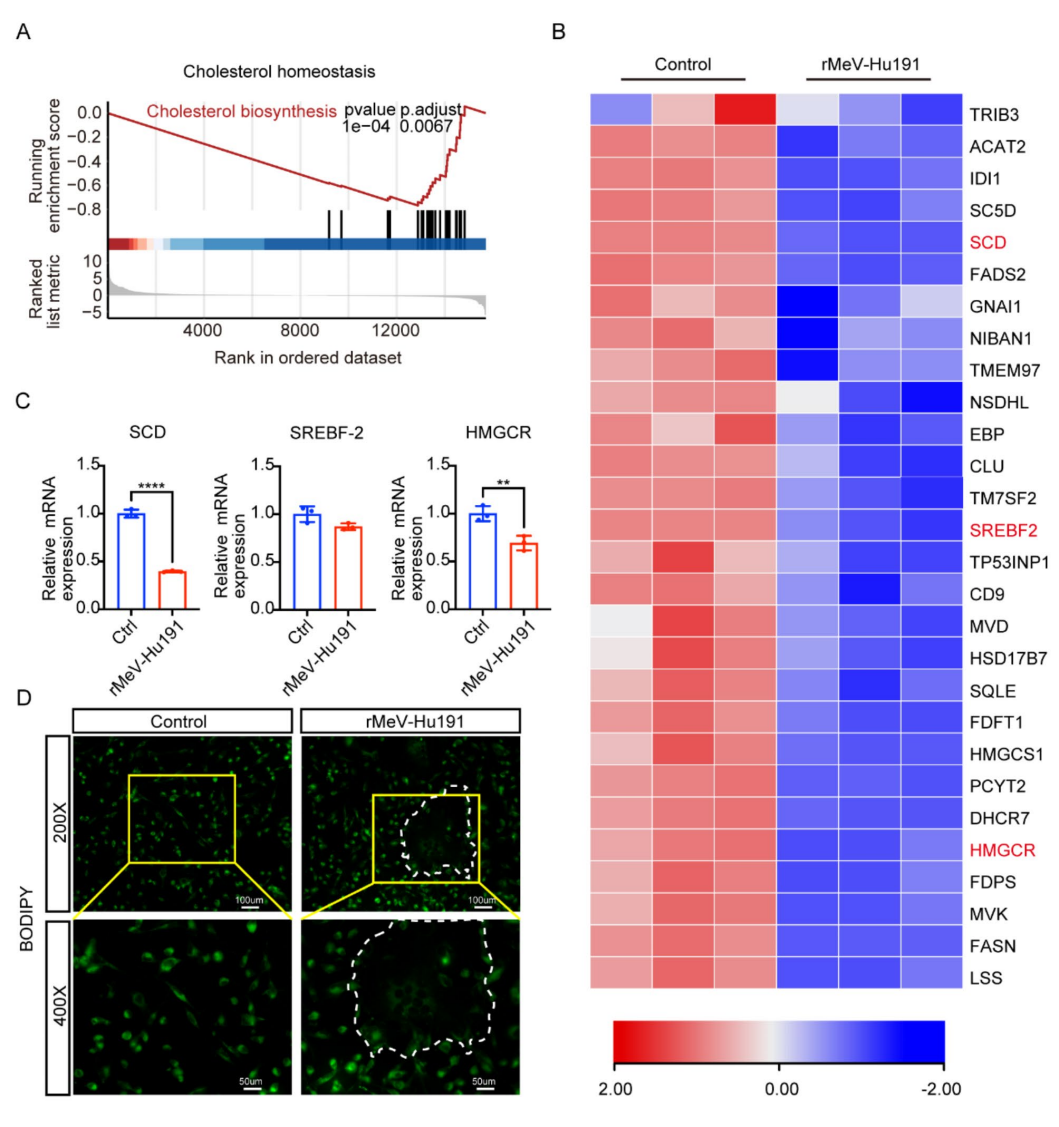
Changes in lipid homeostasis in BC cells following rMeV-Hu191 infection. (**A**) Transcriptome sequencing and gene set enrichment analysis (GSEA) showing the enrichment scores of cholesterol homeostasis-related genes in BC cells after rMeV-Hu191 infection at an MOI of 0.1 for 48 h. (**B**) Heatmap illustrating downregulated genes in control and rMeV-Hu191-infected groups. (**C**) Confirmation of select differential regulated genes (SCD, SREBF-2, and HMGCR) by RT-qPCR, presented as relative mRNA levels. **p < 0.01, ****p < 0.0001. (**D**) BODIPY staining of BC cells infected with rMeV-Hu191 at an MOI of 1 for 24 h, to label lipid content. DAPI indicates nuclear staining

Viral infection affects lipid levels [28, 29]. Therefore, we conducted further analysis to investigate whether rMeV-Hu191 alters lipid homeostasis in BC cells. Transcriptome sequencing and gene set enrichment analysis (GSEA) revealed significant differences in cholesterol biosynthesis between MDA-MB-231 cells with and without rMeV-Hu191 infection (Fig. 4A). The heatmap of differentially regulated genes demonstrated a significant reduction in the mRNA levels of genes associated with cholesterol synthesis and esterification (Fig. 4B). RT-qPCR analysis of selected genes showed that rMeV-Hu191 virus at an MOI of 1 significantly downregulated the relative mRNA levels of SCD (related to fatty acid metabolism) and HMGCR (related to cholesterol synthesis) in BC cells (Fig. 4C). Additionally, BODIPY staining revealed a marked decrease in intracellular lipid droplets in the syncytial region of rMeV-Hu191-infected BC cells (dashed line) (Fig. 4D). These findings confirmed that rMeV-Hu191 infection modulated lipid homeostasis, particularly cholesterol metabolism, in BC cells.

### Oncogenic effects of rMeV-Hu191 in a nude mouse BC model

**Fig. 5:**
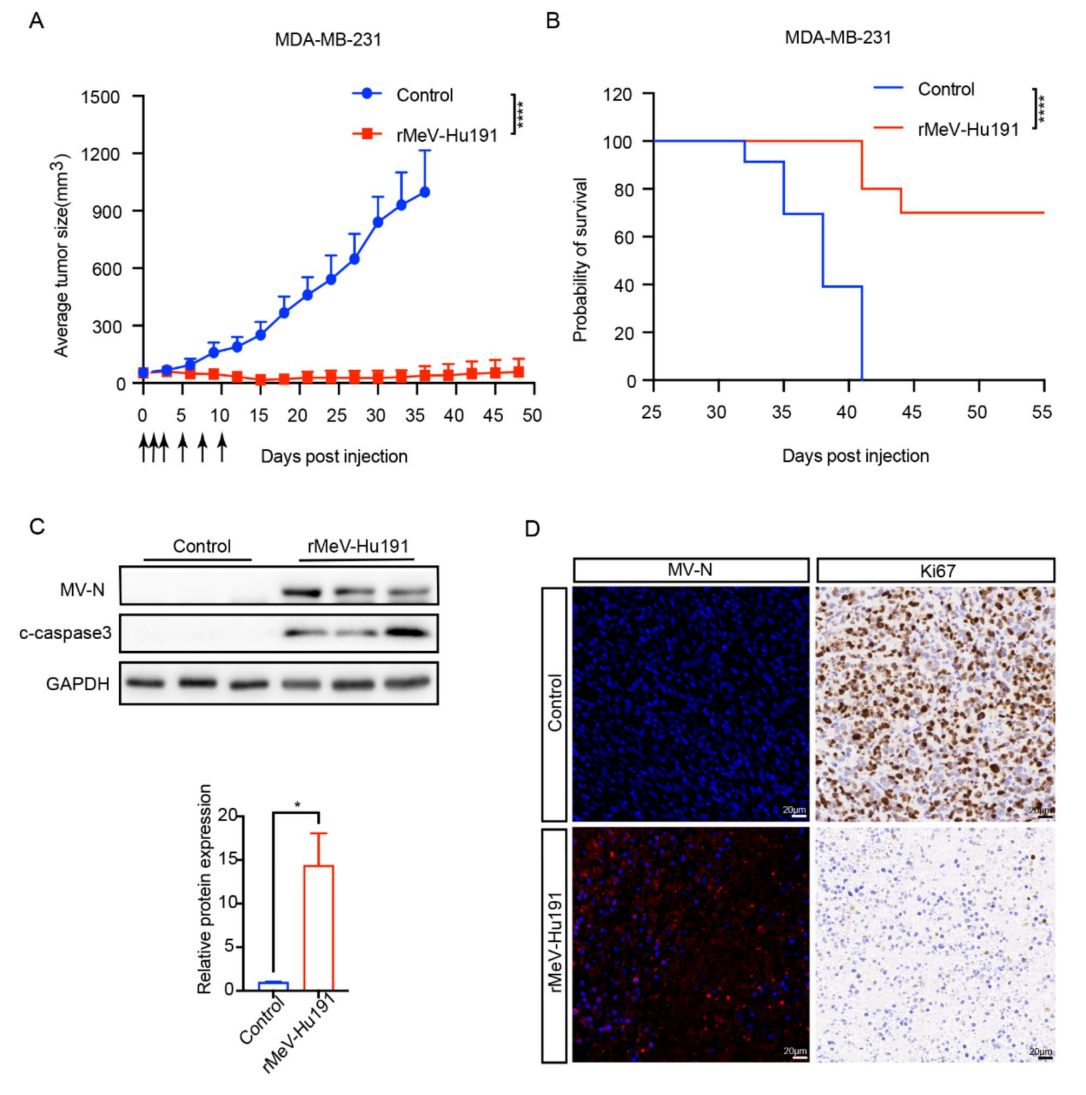
Suppression of BC induced by rMeV-Hu191 in a nude mouse transplanted tumor model. (**A**) Changes in tumor volume over time in the mouse model. The blue line represents the mock-treated control group, while the red line represents the rMeV-Hu191 injection group. ****p < 0.0001. The black arrow indicates the time point of rMeV-Hu191 injection. (**B**) Kaplan–Meier survival curves comparing mice from the rMeV-Hu191 and mock-treated groups using a log-rank (Mantel-Cox) test. ****p < 0.0001. (**C**) Western blot analysis to assess the expression levels of MV-N and cleaved caspase 3 (c-caspase 3) in tissues from the rMeV-Hu191 injection group and the control group. The statistical analysis plots represent the mean ± standard error of three replicate experiments. *p < 0.05. (**D**) Immunofluorescence staining to determine MV-N expression in BC tissues with and without rMeV-Hu191 injection. Immunohistochemistry to analyze the expression of Ki67

Nude mice bearing subcutaneous MDA-MB-231 BC xenografts were used as models. The tumor volume was measured every three days following the injection of 1 × 10^7^ PFU of rMeV-Hu191 into the tumor on the 7th, 8th, 9th, 11th, 13th, and 15th days after tumor implantation. We found that rMeV-Hu191 significantly inhibited BC growth (Fig. 5A) and prolonged survival (Fig. 5B). Western blot analysis of tumor tissue protein levels revealed increased expression of MV-N (a structural protein vital for measles virus replication) and cleaved caspase 3 in the rMeV-Hu191 injection group (Fig. 5C). Immunofluorescence examination of tissue sections from the rMeV-Hu191 injection group showed elevated viral replication, while immunohistochemistry identified weakly positive expression of Ki67 (Fig. 5D). These findings confirmed that rMeV-Hu191 suppressed BC by inhibiting proliferation and inducing apoptosis in an in vivo model.

## Discussion

Our study demonstrated that the measles virus vaccine strain rMeV-Hu191 primarily enters BC cells through the CD40 receptor, leading to oncolytic effects via apoptosis, inhibition of cell proliferation, and induction of senescence. Its mechanisms might be relevant to the regulation of oxidative stress and lipid metabolism. Intratumoral injection of rMeV-Hu191 significantly promoted tumor regression and prolonged the survival of mice in a BC xenograft model. This study provides a foundation for further exploration of rMeV-Hu191 as a potential therapeutic strategy for BC.

The measles virus vaccine strain typically acts by binding to the cell surface receptor CD46 [30], which is often upregulated in many tumor cells [31–38]. Thus, CD46 has emerged as a promising target for oncolytic measles virus therapy [39]. However, targeting CD46 on tumor cells may encounter limitations, as this molecule is expressed in most normal human cells [40]. In addition, there may be variability in CD46 expression even within the same cell line [41]. The wild-type measles-associated receptor, Nectin-4, is a cell adhesion molecule that can be overexpressed in various malignant tumors, including BC [42–45]. Our study showed high expression of both CD46 and Nectin-4 in BC cell lines, particularly CD46, suggesting that the measles virus can effectively target and infect BC cells.

In contrast to previous studies employing the Edmonston measles vaccine strain or genetically engineered wild-type measles virus strains [38], our experiments used rMeV-Hu191, a recombinant vaccine strain developed and rescued by our group based on the measles Hu191 vaccine strain. rMeV-Hu191 exhibited substantial cytotoxic effects on BC cell lines, as evidenced by experiments such as the CCK8 cell viability assay and crystal violet staining. Interestingly, although MDA-MB-231 cells possess fewer types of measles receptors on their cell surface than MDA-MB-468 cells, the former exhibited more pronounced tumor oncolytic effects, suggesting selective differences in rMeV-Hu191-mediated oncolysis among BC cell lines. This phenomenon may be attributed to several factors, including the virus’s replication ability in different BC cell lines, tumor genetic heterogeneity, interferon signaling pathways of the tumor cells, and the antiviral responses of BC cells [46, 47]. Further experiments are required to validate these hypotheses. Our experiments indicated that MDA-MB-468 cells produced more IL-6 after rMeV-Hu191 infection compared to MDA-MB-231 cells (Fig. 3E). Research by Ghazal et al. suggested that IL-6 served as an upstream molecule in the STAT3 signaling pathway, which could promote the proliferation of BC cells [48]. This suggests that having more receptors does not always enhance oncolytic effects, as certain mechanisms can counteract the virus’s oncolytic activity after cellular entry.

Previous studies have reported ROS and oxidative stress play crucial roles in virus-induced oncolysis in cancers by contributing to DNA damage and apoptosis [49, 50]. In addition, ROS exhibits dual effects in BC cells: physiological levels promote tumorigenesis through pathways such as regulating cell proliferation, inducing inflammation, resisting apoptosis, and reprogramming metabolism; whereas elevated ROS levels below the toxic threshold can cause DNA damage, activate proto-oncogenes, and repress oncogenes [51]. Our experiments confirmed that rMeV-Hu191 significantly increased ROS levels and oxidative stress in BC cells during oncolysis, as evidenced by the DCFH-DA and the JC-1 assays.

Moreover, virus-induced apoptosis is a common mechanism involved in the oncolysis of BC [52, 53]. Similarly, we observed that rMeV-Hu191 induced caspase-dependent apoptosis in BC cell lines. This aligns with the notion that elevated oxidative stress can lead to apoptotic cell death, further contributing to the oncolytic effects of the virus.

Our study revealed that rMeV-Hu191 significantly reduced Ki67 expression. Ki67 is a proliferation-associated nuclear protein involved in cell cycle regulation, and it serves as a marker for assessing treatment efficacy and predicting tumor prognosis in BC [54, 55]. We also found that rMeV-Hu191 downregulated CDK4 and cyclin D1 in BC cells. Stephenson and Jen et al. reported that inhibition of CDK4 and cyclin D1 could halt BC growth by inducing cell cycle arrest in the G0/G1 phase [56, 57]. Together with the EdU staining results, we demonstrated that rMeV-Hu191 could inhibit cell proliferation via cell cycle arrest.

When cells are arrested in the cell cycle, pro-growth signaling pathways, such as the mTOR or MAPK pathways, can be activated, driving the cells toward senescence. Cellular senescence leads to stable inhibition of cell growth and serves as a potent antitumor mechanism [58]. Alterations in p21, p16, and lamin B1, among other factors, can lead to the manifestation of senescence-like phenotypes. These proteins play critical roles in regulating cell cycle progression and gene expression. Senescence is also characterized by the secretion of proinflammation cytokines, known as SASP [59, 60]. Fabiana et al. revealed that adenovirus dl922-947 alone could induce senescence in the BC cell lines MDA-MB-231 and MCF-7 [61]. Our study is the first to demonstrate that infection with rMeV-Hu191 can induce a senescence-like phenotype in BC cell lines.

Previous literature has demonstrated a close relationship between virus infection and lipid metabolism, with viruses often enhancing their replication capacity in host cells by altering lipid metabolism [28, 29]. Based on these studies, we further investigated the lipid homeostasis of BC cells after rMeV-Hu191 infection. Enrichment analysis in our study revealed the downregulation of multiple genes related to cholesterol metabolism during the inhibition of BC by rMeV-Hu191. Viruses rely on intracellular cholesterol to maintain their life cycle, thus, most viruses promote cholesterol synthesis [62]. However, the impact on lipid synthesis varies depending on the virus species and the type of cell infected. For example, in Newcastle disease virus-infected DF-1 cells, elevated mRNA levels of genes related to lipogenesis were observed, accompanied by decreased levels of ABCA1 mRNA, which facilitates cholesterol efflux [63]. Conversely, in neuroblastoma cells persistently infected with the measles virus, most genes involved in cholesterol synthesis were downregulated, resulting in decreased cholesterol and lipid rafts in the cell membrane [64]. Our study revealed that rMeV-Hu191 reduced the mRNA levels of the cholesterol synthesis-associated gene HMGCR and the fatty acid metabolism-associated gene SCD in BC cells. Furthermore, it depleted lipids on the cell membrane and reduced the content of intracellular lipid droplets. These findings provide evidence of disrupted lipid homeostasis in BC cells following infection with rMeV-Hu191.

In a nude mouse xenograft model, we confirmed that the rMeV-Hu191 measles vaccine strain had a significant anticancer effect against BC, with apoptosis and proliferation inhibition playing crucial roles in its oncolytic effect.

However, there are some limitations to our study: (1) Although our research identifies several effects of rMeV-Hu191, it does deeply investigate the molecular pathways or interactions underlying these effects. Future studies should aim to explore these mechanisms in greater detail to corroborate and extend our findings. (2) The current study did not extensively address tumor microenvironment (TME) or immunotherapy. OVs can activate the immune system by selectively targeting and destroying cancer cells, releasing tumor antigens, recruiting immune cells to the TME, and initiating a tumor-specific T-cell response [65]. TME can be modified, for example by OVs, to improve the responsiveness of BC cells to immunotherapies such as immune checkpoint inhibitors [66]. However, the TME of BC is highly heterogeneous and can be influenced by inflammatory factors secreted by adipocytes [67, 68]. Future research should focus on analyzing the breast TME through single-cell RNA sequencing, improving OV delivery to the TME via nanotechnology, and using advanced in vitro models to better mimic the TME and assess the oncolytic effects of the virus [69–71].

The ability of rMeV-Hu191 to selectively target and kill cancer cells underscores its clinical potential as a promising addition to current BC treatment options. This engineered measles vaccine strain can be genetically modified to enhance tumor specificity by adding or removing genes, and can be combined with chemotherapy or immunotherapy to synergistically enhance the anti-tumor activity against BC. Future clinical trials will be essential to evaluate the safety, optimal dosage, and therapeutic efficacy of rMeV-Hu191 in humans.

## Conclusions

rMeV-Hu191 exhibits oncolytic activity against BC cells by promoting apoptosis, inhibiting proliferation, and inducing senescence. The underlying mechanisms involve heightened oxidative stress and disrupted lipid metabolism. These findings highlight rMeV-Hu191 as a promising therapeutic approach for BC, offering valuable insights for future clinical applications.

## Electronic supplementary material

Below is the link to the electronic supplementary material.

Supplementary Material 1

Supplementary Material 2

Supplementary Material 3

Supplementary Material 4

## Data Availability

The datasets used or analyzed during the current study are available from the corresponding author upon reasonable request.

## Abbreviations

Annexin V-FITC: Annexin V-fluorescein isothiocyanate
BC: Breast cancer
DCFH-DA: 2’,7’-dichlorodihydrofluorescein diacetate
EdU: 5-ethynyl-2′-deoxyuridine
GSEA: Gene set enrichment analysis
LAMB1: Lamin B1
MMP: Mitochondrial membrane potential
MOI: Multiplicity of infection
MV-N: Measles vitus nucleoprotein
OV: Oncolytic virus
PFU: Plaque-forming units
PI: Propidium iodide
rMeV-Hu191: Measles virus vaccine strain Hu191
RT-qPCR: Reverse transcription quantitative real-time PCR
SASP: Senescence-associated secretory phenotype
SA-β-gal: Senescence-associated β-galactosidase
TNFα: Tumor necrosis factor
WHO: World health organization
