# Supplementary material for: A novel approach for breast cancer treatment: the multifaceted antitumor effects of rMeV-Hu191
Source: Hereditas. 2024 Sep 28;161:36. doi: 10.1186/s41065-024-00337-9 (PMC11439206; doi:10.1186/s41065-024-00337-9)
Supplement: Supplementary file 1 — Supplementary Material 1 [file 41065_2024_337_MOESM1_ESM.docx]

Table S1 Primer sequences used for RT-qPCR analysis

| Target genes | Primer-F | Primer-R |
| --- | --- | --- |
| Human LAMB1 | GATTGCCCAGTTGGAAGCCT | TGGTCTCGTTAATCTCCTCTTCATACA |
| Human p16 | ACCAGAGGCAGTAACCATGC | AAGTTTCCCGAGGTTTCTCA |
| Human p21 | AGCGATGGAACTTCGACTTTG | CGAAGTCACCCTCCAGTGGT |
| Human TNFα | GCCCCCAGAGGGAAGAGTTCCCCA | GCTTGAGGGTTTGCTACAACATGGGC |
| Human IL1b | CTGTCCTGCGTGTTGAAAGA | TTGGGTAATTTTTGGGATCTACA |
| Human IL6 | CCAGGAGCCCAGCTATGAAC | CCCAGGGAGAAGGCAACTG |
| Human IL8 | AAGGAAAACTGGGTGCAGAG | ATTGCATCTGGCAACCCTAC |
| SCD | TCTTCTCTCACGTGGGTTGG | AGCCAGGTTTGTAGTACCTCCT |
| SREBF-2 | GGCAGTCTGGTGGACAATGA | TGGCTCATCTTTGACCTTTGC |
| HMGCR | TGATTGACCTTTCCAGAGCAAG | CTAAAATTGCCATTCCACGAGC |
| Human GAPDH | CCTGTTCGACAGTCAGCCG | CGACCAAATCCGTTGACTCC |
