# Supplementary figures and images for: A novel approach for breast cancer treatment: the multifaceted antitumor effects of rMeV-Hu191
Source: Hereditas. 2024 Sep 28;161:36. doi: 10.1186/s41065-024-00337-9 (PMC11439206; doi:10.1186/s41065-024-00337-9)

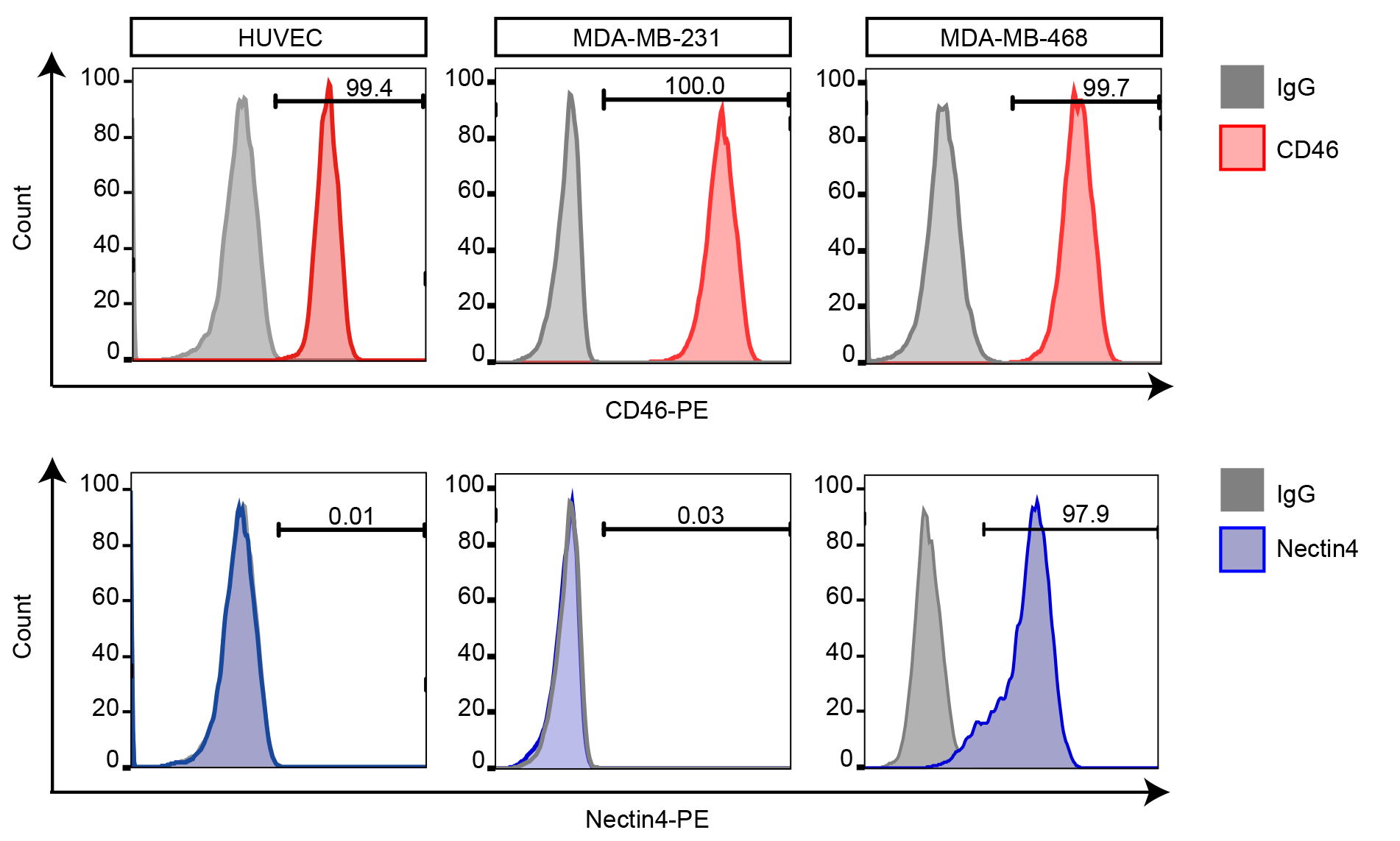

Supplement: Supplementary file 2 — Supplementary Material 2 [file 41065_2024_337_MOESM2_ESM.tif]

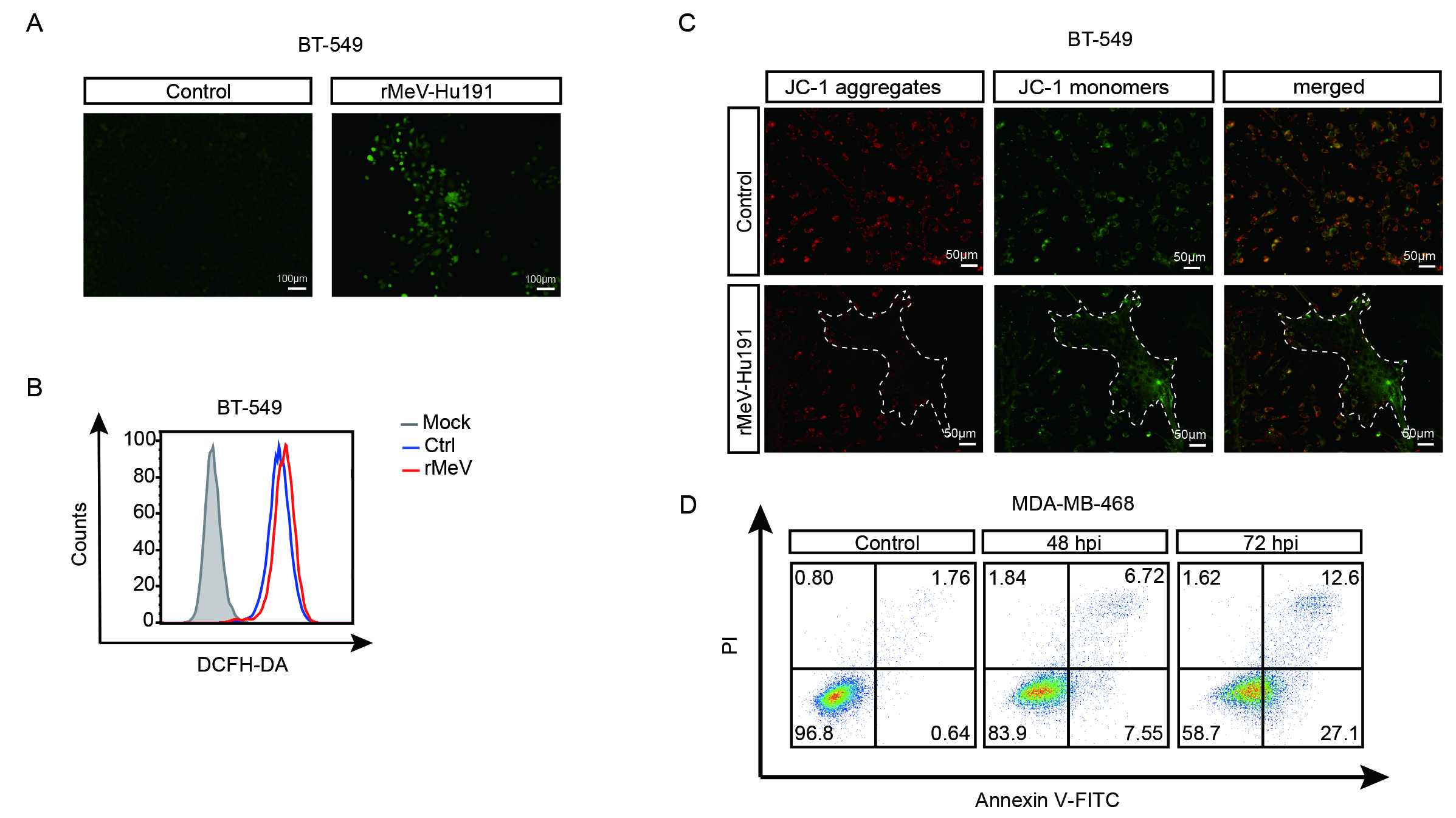

Supplement: Supplementary file 3 — Supplementary Material 3 [file 41065_2024_337_MOESM3_ESM.tif]
