# Supplementary material for: A novel approach for breast cancer treatment: the multifaceted antitumor effects of rMeV-Hu191
Source: Hereditas. 2024 Sep 28;161:36. doi: 10.1186/s41065-024-00337-9 (PMC11439206; doi:10.1186/s41065-024-00337-9)
Supplement: Supplementary file 4 — Supplementary Material 4 [file 41065_2024_337_MOESM4_ESM.docx]

Legend for Supplementary Figure

**Fig. S1** Flow cytometric assay revealing the expression levels of measles virus infection receptor CD46 and Nectin-4 on the cell surface of MDA-MB-231, MDA-MB-468, and HUVEC cells. The HUVEC group served as a control.

**Fig. S2** Oxidative stress damage and apoptosis induced by rMeV-Hu191 in BC cells. (A) Measurement of ROS production in BT-549 cells using 2',7'‐dichlorodihydrofluorescein diacetate (DCFH‐DA) staining. (B) Determination of cellular ROS levels in BT-549 by flow cytometry with DCFH-DA staining. (C) Detection of mitochondrial membrane potential (MMP) changes in rMeV-Hu191-infected BT-549 cells by JC-1 staining. (D) Flow cytometry analysis of apoptotic cells in MDA-MB-468 cells following rMeV-Hu191 infection, as assessed by annexin V-FITC/PI staining.
